# Identifying high-risk Fontan phenotypes using K-means clustering of cardiac magnetic resonance-based dyssynchrony metrics

**DOI:** 10.1016/j.jocmr.2024.101060

**Published:** 2024-07-14

**Authors:** Addison Gearhart, Sunakshi Bassi, Rahul H. Rathod, Rebecca S. Beroukhim, Stuart Lipsitz, Maxwell P. Gold, David M. Harrild, Audrey Dionne, Sunil J. Ghelani

**Affiliations:** a Department of Cardiology, Boston Children’s Hospital, Boston, Massachusetts, USA; b Department of Pediatrics, Harvard Medical School, Boston, Massachusetts, USA; c Division of General Internal Medicine, Brigham and Women's Hospital, Boston, Massachusetts, USA; d Massachusetts Institute of Technology, Boston, Massachusetts, USA; e Department of Cardiology, Children's Hospital of Philadelphia, Phildelphia, Pennsylvannia, USA

**Keywords:** Unsupervised machine learning, Pediatrics, Fontan, Cardiovascular magnetic resonance imaging, Dyssynchrony

## Abstract

**Background:**

Individuals with a Fontan circulation encompass a heterogeneous group with adverse outcomes linked to ventricular dilation, dysfunction, and dyssynchrony. The purpose of this study was to assess if unsupervised machine learning cluster analysis of cardiovascular magnetic resonance (CMR)-derived dyssynchrony metrics can separate ventricles in the Fontan circulation from normal control left ventricles and identify prognostically distinct subgroups within the Fontan cohort.

**Methods:**

This single-center, retrospective study used 503 CMR studies from Fontan patients (median age 15 y) and 42 from age-matched controls from January 2005 to May 2011. Feature tracking on short-axis cine stacks assessed radial and circumferential strain, strain rate, and displacement. Unsupervised K-means clustering was applied to 24 mechanical dyssynchrony metrics derived from these deformation measurements. Clusters were compared for demographic, anatomical, and composite outcomes of death, or heart transplantation.

**Results:**

Four distinct phenotypic clusters were identified. Over a median follow-up of 4.2 y (interquartile ranges 1.7–8.8 y), 58 (11.5%) patients met the composite outcome. The highest-risk cluster (largely comprised of right or mixed ventricular morphology and dilated, dyssynchronous ventricles) exhibited a higher hazard for the composite outcome compared to the lowest-risk cluster while controlling for ventricular morphology (hazard ratio [HR] 6.4; 95% confidence interval [CI] 2.1–19.3; P value 0.001) and higher indexed end-diastolic volume (HR 3.2; 95% CI 1.04–10.0; P value 0.043) per 10 mL/m^2^.

**Conclusion:**

Unsupervised machine learning using CMR-derived dyssynchrony metrics identified four distinct clusters of patients with Fontan circulation and healthy controls with varying clinical characteristics and risk profiles. This technique can be used to guide future studies and identify more homogeneous subsets of patients from an overall heterogeneous population.

## Graphical abstract

**Figure:**
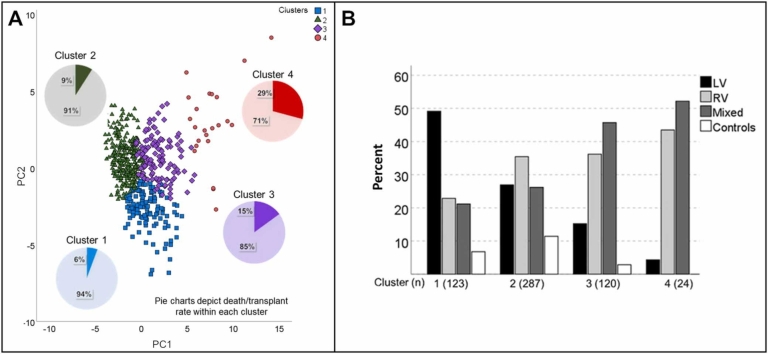

## 1 Introduction

Patients with a functional single ventricle (FSV) palliated to a Fontan circulation are at increasing risk for significant morbidities, heart transplantation, and mortality particularly as they reach adulthood [1], [2]. The wide spectrum of phenotypic and pathophysiological variability poses a challenge in developing effective therapies and managing this population. Cardiovascular magnetic resonance (CMR) feature tracking (FT) can provide detailed information regarding ventricular mechanics by capturing hundreds of variables on both the magnitude and timing of myocardial deformation throughout the cardiac cycle. FT data have been used to characterize mechanical dyssynchrony in FSVs, which has additive prognostic value [3].

Cluster analysis is an unsupervised machine learning technique that can automatically identify subgroups with homogeneous features from heterogeneous datasets. This technique has been shown to offer prognostic utility in adults with heart failure and structurally normal hearts [4]. CMR deformation indices allow for data phenotyping, and superior computational analyses may allow automated classification of repetitive patterns into patient groups with similar behavior (i.e. separate normal ventricular mechanics from disease and identify disease subtypes). A better classification scheme based on ventricular mechanics could potentially improve phenotypic precision and expose potential targeted management strategies in the Fontan population. With this background, the current study hypothesized that unsupervised automated cluster analysis of dyssynchrony metrics could separate ventricles in the Fontan circulation from normal control left ventricles (LV) and identify prognostically distinct subgroups within the Fontan cohort.

## 2 Methods

This was a single-center, retrospective cohort study. The Institutional Review Board approved the study and waived the need for informed consent. Patients with a Fontan circulation who had at least one available CMR between July 1, 2005 and July 1, 2021 were eligible. The oldest analyzable study was included. Ventricular morphology was designated as LV or right ventricle (RV) if the non-dominant ventricle was ≤20% of the combined end-diastolic volume (EDV) and mixed type if the non-dominant ventricle was >20% of the combined EDV. A comparison group was identified as individuals referred for CMR for suspected heart disease but whose studies were subsequently interpreted as normal and did not have known systemic or genetic disease. The comparison group was age-matched ±1 year at the time of the CMR. The ratio of comparison group to patients was ∼1:8 due to the availability of normal CMRs. Demographic and clinical data were extracted from electronic medical records, including the primary end-point of death/heart transplantation.

### 2.1 CMR examinations and feature tracking analysis

The Fontan imaging was conducted according to standard practice, as previously described [5]. Briefly, the endocardial and epicardial borders were manually traced at end-diastole for all slices from the apex to base of the dominant ventricle in the case of a single LV or single RV. For mixed-type ventricles, borders were traced around both ventricles (excluding the ventricular septum). Contours were manually adjusted to ensure optimal tracking. A minimum of four slices was required. The apical slice was defined as the most apical slice with blood pool through the entire cardiac cycle. The basal slice was defined as the most basal slice with a full rim of myocardium through the entire cardiac cycle. For the comparison cohort, the same analysis was performed on the LV. The following conventional measurements were recorded: indexed end-diastolic volume (EDV_*i*_), indexed end-systolic volume (ESV_*i*_), indexed stroke volume, ejection fraction (EF), and indexed ventricular mass. When two ventricles contributed to the systemic circulation, their mass and volumes were combined. For the comparison cohort, only the LV measurements were considered. FT analysis was performed on the short-axis cine stack of images as previously described [3].

### 2.2 Dyssynchrony indices

**Appendix Fig. 1:**
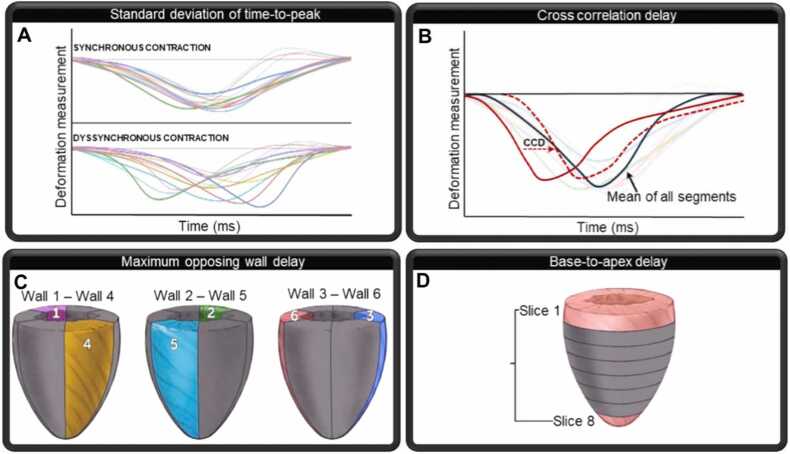
Indices of mechanical dyssynchrony. Four indices of dyssynchrony (A-D) were estimated using segmental data for each of the six deformation measurements (circumferential strain, circumferential strain rate, circumferential displacement, radial strain, radial strain rate, and radial displacement). Segmental data were obtained by dividing each slice into six segments. Cross-correlation delay was obtained for each of the segments compared to the average of all segments.

Six deformation measurements were obtained using FT analysis: circumferential strain, strain rate, and displacement, and radial strain, strain rate, and displacement. Segmental data were obtained by dividing each slice into 6 segments (e.g. in a ventricle with 7 slices, there would be 42 segments). Four dyssynchrony metrics were calculated (Appendix Fig. 1) for each of these six deformation measurements as follows: 1) standard deviation of time-to-peak of each myocardial segment, 2) maximum opposing wall delay as the maximum difference in the average time-to-peak for three opposing segment wall pairs, 3) base-to-apex delay, the difference in the average time-to-peak for the segments in the most basal and most apical slices, and 4) cross-correlation delay (CCD) [3]. CCD is the time shift needed to maximally align a deformation curve to another [6]. CCD was obtained for each of the segments compared to the average of all segments. All images were acquired at 30 phases per cardiac cycle; therefore, each deformation curve had 30 data points (for a heart rate of 100 bpm, each data point would be 20 msec apart). Interpolation was used to preprocess the curves to generate data points at 1 msec intervals. Of all segmental CCD values, the 80th percentile was chosen to represent the degree of global dyssynchrony.

### 2.3 Cluster analysis segregation for dyssynchrony data

K-means clustering was applied to the 24 dyssynchrony metrics standardized to z-scores for the entire cohort (patients and controls). Implementation of the algorithm was performed using Python's scikit-learn library [7]. The optimal number of clusters was determined by the Elbow technique which plots the variance of data explained by the number of proposed clusters and picks the maximum inflection point of the resulting curve as the optimal number of clusters [8]. Principal component analysis was used for dimension reduction for the visual representation of the clusters.

### 2.4 Statistical analysis

Continuous and discrete variables between groups were compared using Mann-Whitney U test and Fisher’s exact test, respectively. Kaplan-Meier survival curves with log-rank test were used to compare the survival between different clusters (controls excluded). From prior work, RV morphology and higher EDV_*i*_ were found to be the strongest independent predictors of outcomes; hence, multivariate Cox regression was performed to assess the added value of cluster designation. Statistical analyses were performed using SAS version 9.4 (SAS Institute, Cary, North Carolina ), SPSS version 27 (IBM Corp, Armonk, New York), and Python version 3.7 (Wilmington, Delaware).

## 3 Results

**Fig. 1:**
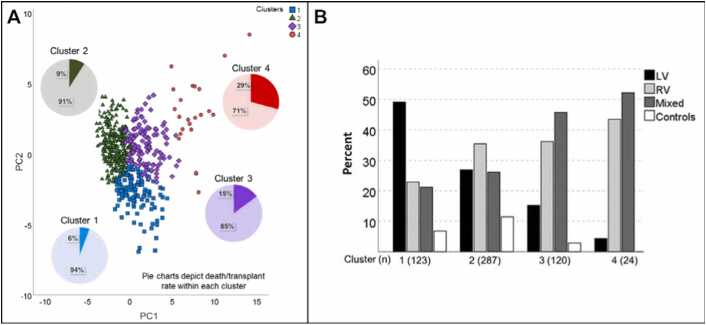
Principal component analysis revealing the distribution of the four clusters and proportion of composite outcome within each cluster (A) and distribution of ventricular morphology within each cluster (B). (A) Principal components are linear combinations of the original dyssynchrony variables in the dataset that capture the total variance of the original dataset and are numbered in decreasing order of importance. The first principal component captures the most variance in the dataset and the second captures the variance orthogonal to the first. Pie charts depict the death/transplant rate within each cluster. (B) Controls represent normal left ventricles while LV, RV, and mixed types represent ventricular morphology for Fontan patients. PC1: principal component 1, PC2: principal component 2, LV: left ventricle, RV: right ventricle.

The cohort included 512 patients (15 y; interquartile ranges [IQR] 10–21 y) and 42 healthy controls (15 y; IQR 11–20 y). Table 1 details the baseline characteristics and CMR variables for the entire cohort. The majority of dyssynchrony metrics were elevated in Fontan patients compared to controls (Table 1, Appendix Table 1). K-means clustering revealed four optimal clusters to describe dyssynchrony data (Fig. 1). Differences between clusters are summarized in Table 2 and Appendix Table 2. The majority of controls were segregated into the lower risk clusters 1 and 2, which also included a higher proportion of single ventricles of LV morphology with more synchronous contraction patterns than the higher risk clusters 3 and 4 (Table 2; Fig. 1). Between the two lowest-risk clusters, cluster 2 tended to be younger with accordingly lower QRS duration and indices of dyssynchrony. Cluster 4 represented patients with dilated ventricles with higher degree of dysfunction and dyssynchrony and had no controls.

**Table 1:** Baseline characteristics, conventional CMR data, and dyssynchrony measurements for all patients and comparison LVs.

	Fontan patients (N = 512)	Comparison LV (N = 42)	P value
*Baseline characteristics*			
Age at CMR, y	15.2 (10.3, 21.3)	15.7 (11.0, 19.7)	0.923
Sex, male	302 (60%)	18 (43%)	0.030*
BSA, m^2^	1.5 (1.1, 1.8)	1.6 (1.3, 1.8)	0.044*
SBP, mmHg	113 (101,123)	117 (106, 124)	0.239
SBP Z-score	0.26 (−0.62, 1.11)	0.50 (−0.26, 1.20)	0.287
Heart rate, bpm	82 (70, 93)	80 (68, 89)	0.628
QRS interval, ms	100 (90, 116)	84 (78, 92)	<0.001*
**Cardiac diagnosis**			
HLHS	147 (29%)		
Tricuspid atresia	83 (16%)		
DORV	63 (12%)		
DILV	57 (11%)		
Complex 2V	54 (11%)		
CAVC	44 (9%)		
Hypoplastic RV/TV	26 (5%)		
PAIVS	24 (5%)		
Mitral atresia	14 (3%)		
*Conventional CMR variables*			
EDV_*i*_, mL/BSA (N = 467)	107 (92, 134)	82 (73, 93)	<0.001*
ESV_*i*_, mL/BSA (N = 467)	50 (39, 68)	32 (27, 38)	<0.001*
Mass_*i*_, grams/BSA (N = 449)	55 (46, 70)	48 (42, 54)	<0.001*
EF % (N = 467)	54 (47, 58)	60 (56, 66)	<0.001*
Indexed stroke volume (N = 467)	57 (48, 66)	51 (44, 55)	0.003*
*Dyssynchrony metrics*			
GCS (%)	−15 (−17, −12)	−18 (−20, −17)	<0.001*
SDTTP CS (ms)	51 (40, 71)	45 (37, 52)	0.01*
MOWD CS (ms)	56 (38, 81)	42 (31, 49)	0.001*
BAD CS (ms)	27 (15, 46)	25 (12, 39)	0.25
CCD CS (ms)	41 (31, 55)	28 (23, 38)	<0.001*
GRS (%)	22 (18, 27)	29 (27, 34)	<0.001*
STD TTP RS (ms)	51 (38, 70)	44 (36, 53)	0.036*
MOWD RS (ms)	54 (35, 77)	39 (28, 49)	<0.001*
BAD RS (ms)	27 (15, 43)	22 (12, 39)	0.215
CCD RS (ms)	39 (31, 53)	28 (23, 35)	<0.001*

Values are medians (interquartile range) or counts (%).

*CMR* cardiovascular magnetic resonance, *BSA* body surface area, *SBP* systolic blood pressure, *HLHS* hypoplastic left heart syndrome, *DORV* double outlet right ventricle, *DILV* double inlet left ventricle, *2V* two ventricles, *CAVC* complete atrioventricular canal defect, *RV* right ventricle, *TV* tricuspid valve, *PAIVS* pulmonary atresia intact ventricular septum, *EDV_i_* indexed ventricular end-diastolic volume, *ESV_i_* indexed ventricular end-systolic volume, *Mass_i_* indexed ventricular mass, *EF* ejection fraction, *GCS* global circumferential strain, *SDTTP* standard deviation of time-to-peak of all segments, *CS* circumferential strain, *MOWD* maximum opposing wall delay, *BAD* base-to-apex delay, *CCD* cross-correlation delay, *GRS* global radial strain, *RS* radial strain, *LV* left ventricle.

* P value <0.05 comparing the entire Fontan cohort and the comparison LVs.

**Table 2:** Comparison of demographics, conventional CMR data, and dyssynchrony measurements between clusters.

	Cluster				P value (*<0.05, **≤0.01)					
	1 (N = 123)	2 (N = 287)	3 (N = 120)	4 (N = 24)	1 vs 2	1 vs 3	1 vs 4	2 vs 3	2 vs 4	3 vs 4
Age at CMR, y	17.5 (12.8, 23.5)	12.5 (8.5, 17.9)	17.6 (12.7, 23.5)	16.7 (11.3, 20.1)	**	NS	NS	**	*	NS
Sex, male	73 (59%)	152 (53%)	79 (66%)	20 (83%)	NS	NS	NS	*	*	NS
BSA, m^2^	1.6 (1.3, 1.8)	1.3 (0.9, 1.7)	1.6 (1.2, 1.8)	1.5 (1.1, 1.7)	**	NS	NS	**	*	NS
SBP, mmHg	113 (105, 121)	111 (97, 121)	119 (106, 129)	118 (114, 125)	NS	NS	NS	**	*	NS
Heart rate, bpm	63 (56, 73)	86 (75, 98)	75 (65, 86)	74 (60, 88)	**	**	*	**	**	NS
QRS duration, ms	101 (92, 114)	94 (86, 107)	106 (92, 120)	122 (106, 135)	**	NS	**	**	**	**
EDV_*i*_ (mL/m^2^)	101 (87, 121)	101 (85, 127)	111 (98, 145)	169 (126, 270_	NS	**	**	**	**	**
ESV_*i*_ (mL/m^2^)	54 (47, 65)	45 (35, 62)	56 (41, 78)	109 (64, 183)	NS	**	**	**	**	**
Mass_*i*_	52 (44, 65)	49 (42, 61)	57 (44, 72)	75 (61, 93)	NS	NS	**	**	**	**
EF (%)	56 (51, 61)	56 (50, 59)	51 (41, 58)	34 (28, 47)	NS	**	**	**	**	**
SV_*i*_ (mL/m^2^) (N = 467)	54 (47, 65)	55 (47, 64)	59 (50, 72)	60 (48, 71)	NS	NS	NS	NS	NS	NS
*Dyssynchrony*										
GCS (%)	−16 (−18, −15)	−16 (−18, −13)	−13 (−15, 11)	−8 (−10, −6)	NS	**	**	**	**	**
SDTTP GCS (ms)	48 (43, 64)	43(34, 54)	77 (62, 98)	132 (110, 152)	**	**	**	**	**	**
MOWD CS (ms)	52 (28, 79)	44 (33,61)	78 (54, 114)	128 (72, 166)	**	**	**	**	**	**
BAD CS (ms)	37 (29, 69)	28 (18, 55)	51 (27, 85)	68 (27, 93)	**	NS	NS	**	**	NS
CCD CS (ms)	45 (36, 52)	33 (27, 40)	59 (45, 78)	150 (122, 210)	**	**	**	**	**	**
GRS (%)	26 (22, 29)	24 (19, 30)	18 (15, 22)	11 (7,15)	**	**	**	**	**	**
SDTTP RS (ms)	48 (41, 62)	42 (34, 52)	75 (59, 93)	116 (107, 146)	**	**	**	**	**	**
MOWD RS (ms)	51 (35, 78)	44 (31, 60)	72 (52, 107)	114 (64, 162)	**	**	**	**	**	**
BAD GRS (ms)	36 (25, 66)	28 (18, 56)	48 (24, 81)	46 (26, 93)	*	NS	NS	**	**	NS
CCD GRS (ms)	42 (35, 51)	33 (26, 38)	57 (41, 70)	139 (112, 170)	**	**	**	**	**	**

Values are medians (interquartile range) or counts (%).

*CMR* cardiovascular magnetic resonance, *BSA* body surface area, *SBP* systolic blood pressure, *HLHS* hypoplastic left heart syndrome, *DORV* double outlet right ventricle, *DILV* double inlet left ventricle, *2V* two ventricles, *CAVC* complete atrioventricular canal defect, *RV* right ventricle, *TV* tricuspid valve, *PAIVS* pulmonary atresia intact ventricular septum, *EDV_i_* indexed ventricular end-diastolic volume, *ESV_i_* indexed ventricular end-systolic volume, *Mass_i_* indexed ventricular mass, *SV_i_* indexed stroke volume, *EF* ejection fraction, *GCS* global circumferential strain, *SDTTP* standard deviation of time-to-peak of all segments, *CS* circumferential strain, *MOWD* maximum opposing wall delay, *BAD* base-to-apex delay, *CCD* cross-correlation delay, *GRS* global radial strain, *RS* radial strain.

* P < 0.05.

** P ≤ 0.01.

**Fig. 2:**
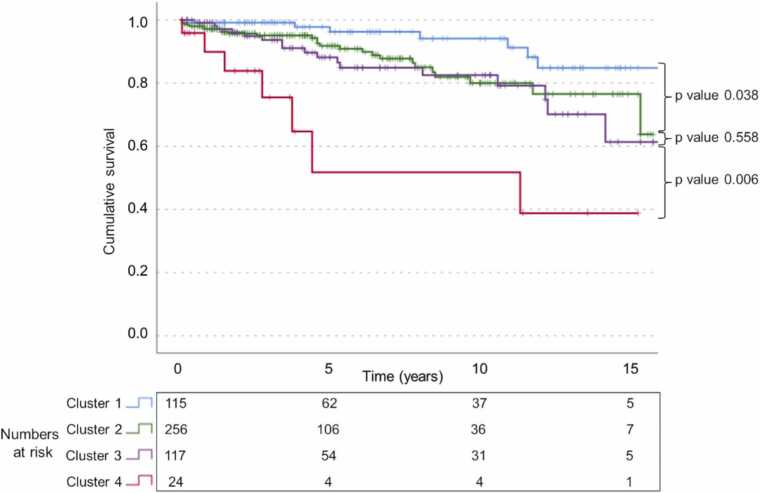
Kaplan-Meier survival curves for freedom from death or heart transplantation/listing from time to CMR stratified by cluster. Patients in cluster 1 experienced the greatest freedom from the composite outcome. Patients in cluster 4 had the lowest freedom from the composite outcome. P values are from pairwise log-rank tests. *CMR*, cardiovascular magnetic resonance

Over a median follow-up of 4.2 y (IQR 1.7 y, 8.8 y), 58 (11.5%) patients had the composite outcome. Cluster 1 had the highest survival, cluster 4 had lowest survival, and clusters 2 and 3 were similar. Multivariate Cox regression showed that cluster 4 carried an increased HR of the composite outcome while controlling for ventricular morphology (hazard ratio [HR] for cluster 4 of 6.4 (95% confidence interval [CI] 2.1–19.3), 3.2 (95% CI 1.4–7.6), and 3.2 (95% CI 1.3–8.1) compared to clusters 1, 2, and 3, respectively); or EDV_*i*_ (HR for cluster 4 of 3.2 (95% CI 1.04–10.0), 1.6 (95% CI 0.67–4.0), and 1.4 (95% CI 0.5–3.7) compared to clusters 1, 2, and 3, respectively; Appendix Table 3). Fig. 2 depicts Kaplan-Meier freedom from the outcome of death or heart transplantation for clusters.

## 4 Discussion

This study demonstrated that an unsupervised machine learning approach, based on the input of CMR-derived metrics of dyssynchrony alone, can identify distinct clusters of subjects within a heterogeneous population of controls and FSVs. The lower risk clusters 1 and 2 contained the majority of the controls and single LVs with more synchronous contraction patterns compared to the higher risk clusters 3 and 4. Clusters corresponded to increasing risk for death or heart transplantation or transplantation listing in the Fontan population and the prognostic implication of a cluster assignment was independent of established risk factors.

The application of clustering analyses to cardiovascular disease has been confined primarily to adults and the analysis of multiple clinical variables, biomarker characteristics, and echocardiographic markers of diastolic dysfunction and global strain [4], [9]. The novel concept of this work was the inclusion of healthy controls and the sole input data of dyssynchrony metrics. The clusters do not perfectly separate out the controls from FSVs; however, the separation is revealing. It identified a cluster with no controls that had the highest risk of death or heart transplantation or heart transplantation listing. Between the two lowest-risk clusters, patients in the lower risk cluster 1 were older at CMR and had more patients with single LV morphology than cluster 2. They also exhibited higher event-free survival even in the setting of longer QRS duration and more ventricular dyssynchrony. Taken together, this suggests single LV morphology may have a protective effect even in the setting of mechanical dyssynchrony.

Developing a better understanding and classification scheme for Fontan patients could allow for more focused clinical trials of disease-modifying therapies (i.e. enabling enrollment and surveillance of a homogeneous group of patients for cardiac resynchronization therapy). Although multicenter collaboration with larger datasets and external validation isolated to just patients with a Fontan circulation should precede such clinical application, the current study provides preliminary data to plan future studies.

Other than EF, few indices of ventricular deformation have been used in the risk stratification of FSVs [10]. A number of metrics for capturing dyssynchrony have been proposed but there is no gold standard or proven metric. The current study introduced a new metric of dyssynchrony, CCD. It also reaffirmed that RV morphology and ventricular dilation are associated with death or heart transplantation in the Fontan population. Cluster 4 had a significantly increased hazard of the composite outcome even after controlling for ventricular dilation and morphology, implying that cluster assignment holds value beyond established risk factors.

This study should be viewed as a proof-of-concept and results should be seen as hypothesis-generating and warrant replication with an external dataset. As such, the limitations of K-means clustering and the potential for overfitting data should be acknowledged. It is subject to selection bias as patients referred for CMR are likely to be older and potentially sicker. The benefit of clustering patients beyond known risk factors has yet to be fully determined. Given the relatively low rate of outcomes, our prediction model focused on exploring if clustering assignment was additive beyond the well-established risk factors.

## 5 Conclusions

Unsupervised machine learning using CMR-derived dyssynchrony metrics alone identified four distinct clusters of patients with Fontan circulation and healthy controls with varying clinical characteristics and risk profiles. In the future, this technique could be used to guide future studies and identify more homogeneous subsets of patients from an overall heterogeneous population who may benefit from targeted therapies.

## Funding

A.G. is supported by Matthew’s Heart of Hopes Grant and 10.13039/100000002NIH T32 grant. There are no relationships with the industry to disclose.

## Author contributions

**Audrey Dionne:** Writing – review and editing, Conceptualization. **Sunil J. Ghelani:** Writing – review and editing, Writing – original draft, Visualization, Resources, Project administration, Methodology, Formal analysis, Data curation, Conceptualization. **Addison Gearhart:** Writing – review and editing, Writing – original draft, Methodology, Data curation, Conceptualization. **Sunakshi Bassi:** Conceptualization. **Rahul H. Rathod:** Data curation, Conceptualization. **Rebecca S. Beroukhim:** Writing – review and editing, Writing – original draft. **Stuart Lipsitz:** Formal analysis, Conceptualization. **Maxwell P. Gold:** Visualization, Validation, Software, Methodology, Formal analysis, Data curation, Conceptualization. **David M. Harrild:** Methodology, Conceptualization.

## Ethics approval and consent

This article has achieved Institutional Review Board approval.

## Declaration of Competing Interest

The authors declare that they have no known competing financial interests or personal relationships that could have appeared to influence the work reported in this paper.

## Figures and Tables

**Appendix Table 1:** Remaining dyssynchrony metrics for all Fontan patients vs comparison left ventricles.

	Fontan (N = 512)	Controls (N = 42)	P value
SDTTP CSR (ms)	116 (86, 151)	82 (59, 115)	<0.001**
MOWD CSR (ms)	105 (72, 152)	61 (43, 92)	<0.001**
BAD CSR (ms)	60 (23, 139)	30 (0, 59)	0.003**
CCD CSR (ms)	27 (17, 44)	24 (19, 30)	0.110
SDTTP RSR (ms)	96 (75, 122)	75 (59, 99)	<0.001**
MOWD RSR (ms)	83 (53, 130)	61 (40, 81)	<0.001**
BAD RSR (ms)	38 (19, 84)	31 (0, 67)	0.164
CCD RSR (ms)	36 (24, 53)	25 (21, 37)	<0.001**
SDTTP CD (ms)	107 (90, 125)	119 (106, 134)	<0.001**
MOWD CD (ms)	98 (67, 129)	95 (69, 135)	0.930
BAD CD (ms)	92 (41, 169)	115 (69, 135)	0.329
CCD CD (ms)	281 (193, 369)	178 (126, 252)	<0.001**
SDTTP RD (ms)	61 (48, 76)	49 (38, 60)	<0.001**
MOWD RD (ms)	69 (45, 100)	59 (40, 79)	0.092
BAD RD (ms)	47 (23, 72)	29 (16, 53)	0.039*
CCD RD (ms)	58 (44, 74)	38 (32, 49)	<0.001**

Values are medians (interquartile range) or counts (%).

*SDTTP* standard deviation of time-to-peak of all segments, *CSR* circumferential strain rate, *MOWD* maximum opposing wall delay, *BAD* base to apex delay, *CCD* cross-correlation delay, *RSR* radial strain rate, *CD* circumferential displacement, *RD* radial displacement.

* P < 0.05.

** P ≤ 0.01.

**Appendix Table 2:** Remaining dyssynchrony measurements compared across clusters.

	Cluster				P value (*<0.05 and **≤0.01)					
	1 (N = 123)	2 (N = 287)	3 (N = 120)	4 (N = 24)	1 vs 2	1 vs 3	1 vs 4	2 vs 3	2 vs 4	3 vs 4
SDTTP CSR (ms)	104 (76,139)	174 (151, 207)	92 (79, 118)	116 (107, 145)	**	**	**	**	**	NS
MOWD CSR (ms)	103 (67, 133)	84 (60, 130)	137 (97, 208)	197 (120, 257)	**	**	**	**	**	NS
BAD CSR (ms)	59 (28, 118)	44 (21, 116)	102 (27, 174)	137 (22, 210)	**	**	*	**	*	NS
CCD CSR (ms)	29 (21, 38)	21 (14, 29	48 (30, 68)	131 (111, 164)	**	**	**	**	**	**
SDTTP RSR (ms)	82 (71, 100)	86 (67, 104)	128 (106, 154)	163 (142, 195)	NS	**	**	**	**	**
MOWD RSR (ms)	75 (50, 116)	67 (44, 98)	111 (80, 176)	178 (139, 234)	NS	**	**	**	**	**
BAD RSR (ms)	34 (0, 69)	30 (16, 67)	55 (26, 134)	70 (24, 156)	NS	**	NS	**	*	NS
CCD RSR (ms)	36 (28, 48)	26 (20, 38)	56 (39, 75)	130 (112, 162)	**	**	**	**	**	**
SDTTP CD (ms)	123 (106, 143)	100 (86, 113)	116 (95, 135)	114 (92, 128)	**	*	NS	**	*	NS
MOWD CD (ms)	105 (77, 151)	89 (66, 117)	106 (64, 135)	91(70, 149)	**	NS	NS	*	NS	NS
BAD CD (ms)	102 (35, 187)	88 (40, 155)	106 (50, 175)	153 (37, 217)	NS	NS	NS	NS	NS	NS
CCD CD (ms)	336 (208, 499)	249 (157, 320)	293 (202, 384)	322 (216, 392)	**	NS	NS	**	*	NS
STD RD (ms)	58 (48, 71)	52 (42, 66)	72 (61, 87)	97 (73, 118)	**	**	**	**	**	**
MOWD RD (ms)	74 (45, 105)	58 (39, 83)	84 (56, 118)	100 (69, 2)	**	NS	**	**	**	NS
BAD RD (ms)	43 (28, 74)	41 (21, 66)	57 (27, 88)	51 (26, 97)	NS	NS	NS	**	NS	NS
CCD RD (ms)	58 (48, 71)	48 (36, 62)	69 (58, 88)	117 (64, 159)	**	**	**	**	**	**

Values are medians (interquartile range) or counts (%).

*NS* not statistically significant, *SDTTP* standard deviation of time-to-peak of all segments, *CSR* circumferential strain rate, *MOWD* maximum opposing wall delay, *BAD* base to apex delay, *CCD* cross-correlation delay, *RSR* radial strain rate, *CD* circumferential displacement, *RD* radial displacement.

* P < 0.05.

** P < 0.01.

**Appendix Table 3:** Cox regression analysis using time from CMR to composite outcome for cluster assignment isolated to Fontan patients while controlling for ventricular morphology (model 1) and EDV_*i*_ (model 2).

Model 1 (N = 512)			Model 2 (N = 512)		
	HR (95% CI)	P value		HR (95% CI)	P value
RV (index: LV)	2.3 (1.1–5.0)	0.034	EDV_*i*_ (per 10 mL/m^2^)	1.1 (1.06–1.14)	<0.001
Mixed	0.56 (0.25–1.26)	0.160			
Cluster 4 vs 1	6.4 (2.1–19.3)	0.001	Cluster 4 vs 1	3.2 (1.04–10.0)	0.043
Cluster 4 vs 2	3.2 (1.4–7.6)	0.008	Cluster 4 vs 2	1.6 (0.67–4.0)	0.251
Cluster 4 vs 3	3.2 (1.3–8.1)	0.012	Cluster 4 vs 3	1.4 (0.5–3.7)	0.270

*RV* right ventricle, *LV* left ventricle, *EDV_i_* indexed end-diastolic volume, *HR* hazard ratio, *CI* confidence interval.

## Data Availability

Data are available upon request. The code for the unsupervised machine learning technique used is available upon request.
